# Recurrent Grade I Parafalcine Meningioma: Management Challenges and Genomic Insights

**DOI:** 10.7759/cureus.89466

**Published:** 2025-08-06

**Authors:** Jenna R Rocchetti, Nilanjan Haldar, Debanjan Haldar, David W Andrews, Maria Werner-Wasik

**Affiliations:** 1 Radiation Oncology, Thomas Jefferson University Hospital, Philadelphia, USA; 2 Neurosurgery, Thomas Jefferson University Hospital, Philadelphia, USA

**Keywords:** central nervous system, craniotomy, meningioma, parafalcine meningioma, radiation oncology, radiosurgery

## Abstract

While World Health Organization (WHO) grade I meningiomas are typically slow growing and associated with favorable prognoses, a subset may exhibit unexpectedly aggressive behavior and resistance to conventional treatment approaches. Recurrent grade I meningiomas, in particular, are associated with a poorer prognosis despite their benign histological classification, underscoring the need for advanced genomic and radiomic analyses to refine diagnostic accuracy. We present a case of a 52-year-old female with a grade I parafalcine meningioma initially deemed nonaggressive, but ultimately recurred multiple times over several years despite undergoing repeated craniotomies and several courses of radiosurgery. Notable tumor characteristics included its supratentorial location, high Ki-67 proliferative index, and NF-2 genetic alteration, which are all factors associated with decreased progression-free survival in grade I meningioma patients. The patient remains clinically stable eight months post salvage stereotactic radiation therapy (SRT). This case highlights the intricate surgical and radiotherapeutic decisions involved in managing recurrent grade I meningiomas, reinforcing the importance of multidisciplinary evaluation and individualized treatment strategies.

## Introduction

Meningiomas constitute the most common primary tumors of the central nervous system (CNS), accounting for approximately one-third of intracranial neoplasms [1]. Although typically classified as nonaggressive, meningiomas present with a range of symptoms depending on the anatomical location of the primary tumor and degree of compression on adjacent structures [1]. Further, grade I meningiomas comprise 80.5% of all meningioma cases and are generally characterized by a benign and indolent clinical course [2].

Management of symptomatic meningiomas primarily involves surgical resection, with adjuvant radiation therapy (RT), if necessary [1]. Although gross total resection (GTR) of grade I meningiomas generally results in a favorable prognosis, tumors in challenging locations may only achieve subtotal resection (STR), leading to worse outcomes regarding local control. For example, although tumors within the parasagittal region are superficial, they are associated with high rates of surgical complications, such as air embolism, significant blood loss, or postoperative sinus thrombosis, because these tumors grow along the line of the superior sagittal sinus, leading to tumor invasion into the sinus [3]. Parafalcine meningiomas, which originate from the falx cerebri and are located within the interhemispheric fissure, have the third highest morbidity among all meningiomas, accounting for 11-14% of deaths, right behind parasagittal and cerebral convexity meningiomas [4]. Additionally, 34.8% of parafalcine and parasagittal meningiomas have postoperative complications, including mutism and hemiparesis due to injury of the supplementary motor cortex, cingulated cortex, and corpus callosum [5]. As 30% of meningiomas are parafalcine and parasagittal in location, and a large majority achieve only STR, adjuvant therapy is often indicated [6].

Recurrent grade I meningiomas, in sharp contrast to initially diagnosed tumors, have a significantly poorer prognosis clinically and are treated as a different entity. This is due to numerous reasons, including their propensity to undergo malignant transformations, ultimately becoming more aggressive and difficult to control. Studies indicate that malignant transformation in grade I meningiomas occurs at rates ranging from 0.16% to 2.0% [7]. Further research is ongoing to characterize these meningiomas and identify what puts patients at risk for transformation.

In the management of recurrent or subtotally resected grade I meningiomas, stereotactic radiosurgery (SRS) or fractionated radiotherapy (FRT) is indicated [8]. For tumors measuring ≤3cm in diameter or 10 cm^3^ in volume, SRS (12-16 Gy single fraction) is typically preferred, whereas FRT (50-55 Gy delivered in 1.8-2.0 Gy fractions) is utilized when the tumor volume cannot be treated with a single fraction [8].

In contrast, higher-grade meningiomas (grades II and III) have recurrence rates of 30-40% and 50-80% after five years, respectively [8]. Therefore, they undergo adjuvant FRT (54-60Gy in 1.8-2.0 Gy/fraction) after surgery, regardless of surgical outcomes [8]. It is important to note, however, that there are challenges with reirradiation of these sensitive tumors, and radiation toxicity can become a limiting factor in carrying out the proper treatment plan. Interestingly, recent studies indicate that hypofractionated SRS can decrease complications, such as edema and radiation necrosis in tumors >10 mm^3^, allowing for normal tissues to heal between treatments, thereby reducing radiation toxicity [3].

Herein, we present a case of a recurrent grade I parafalcine meningioma, initially deemed nonaggressive, highlighting the intricate surgical and radiotherapeutic decisions involved in its management.

This article was previously presented as a meeting poster at the ACRO 2025 Summit on March 13, 2025.

## Case presentation

Our patient was a 52-year-old female diagnosed with a grade I recurrent parafalcine vertex meningioma. She initially underwent a craniotomy for tumor resection at an outside hospital, but 10 years post surgery, a recurrence of a left frontal meningioma was identified, occurring just anterior to the previous craniotomy site. The risks associated with single-fraction radiosurgery versus surgical re-excision were evaluated, and due to the potential adverse effects of radiation, including edema and seizures, surgical resection was chosen. The patient, therefore, underwent a second craniotomy, with intraoperative monitoring utilizing motor-evoked and somatosensory-evoked potentials. Postoperative exam was unremarkable with no neurological deficits noted. Pathology revealed a grade I meningioma, with a Ki-67 index of 1.3%.

Postoperative imaging revealed no evidence of recurrence of this recurrent left parietal meningioma. However, two years later, magnetic resonance imaging (MRI) indicated a clear increase in the size of this parietal mass, representing progression in her left parasagittal meningioma. Following discussion at the tumor board, fractionated linear accelerator (LINAC)-based SRS was recommended, delivering 5400 cGy of stereotactic radiation therapy (SRT) in 30 fractions. Despite post-treatment symptoms of headaches and scalp discomfort, MRI follow-up demonstrated stable disease.

Two years later, the patient reported dizziness and lightheadedness again. Scans showed stable disease within the treatment field with a new 7 mm recurrent lesion adjacent to the primary tumor. Biopsy found this to be a new small left parietal meningioma, representing a geographic miss in therapy. The multidisciplinary decision was made with radiation oncology and neurosurgical teams to recommend retreatment with single-fraction LINAC radiosurgery. The patient received 1500 cGy in one fraction with no radiation toxicity noted. Follow-up brain MRIs showed no evidence of disease progression of the parafalcine meningioma. The lateral meningioma adjacent to this primary tumor also reduced in volume.

**Figure 1:**
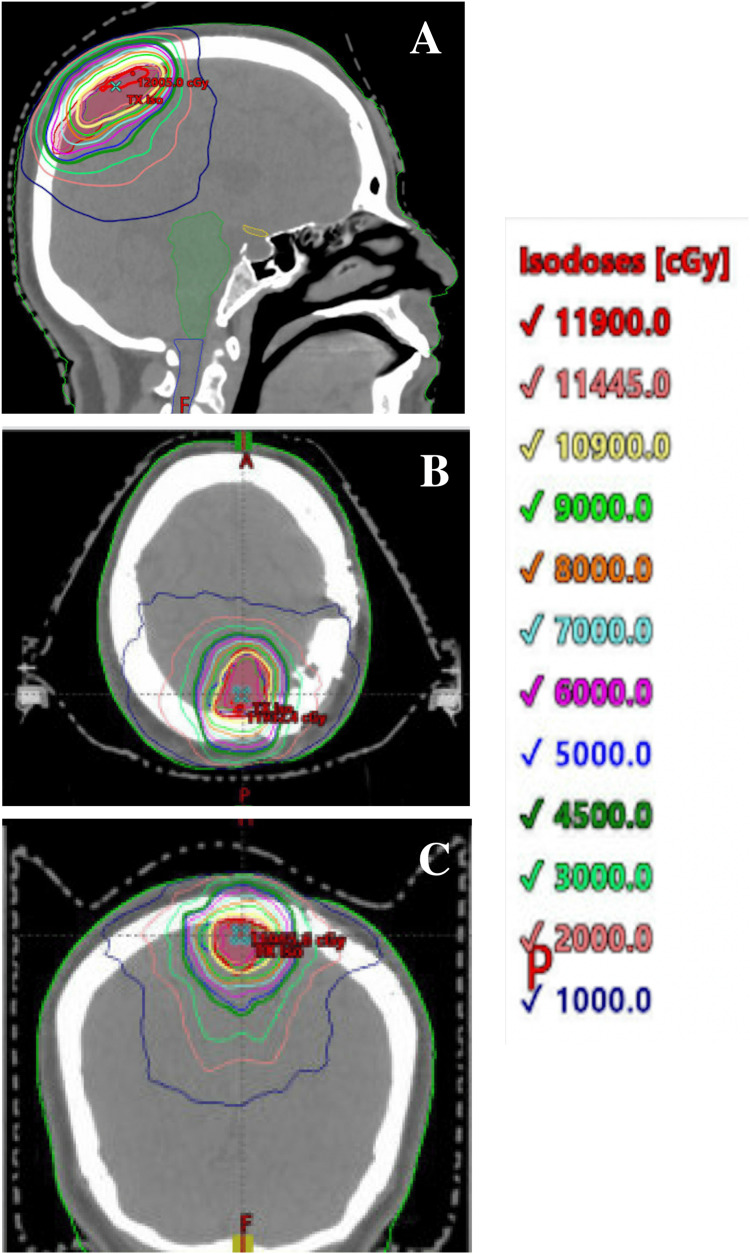
Composite dose with isodose lines in (A) sagittal, (B) transverse, and (C) frontal planes.

**Figure 2:**
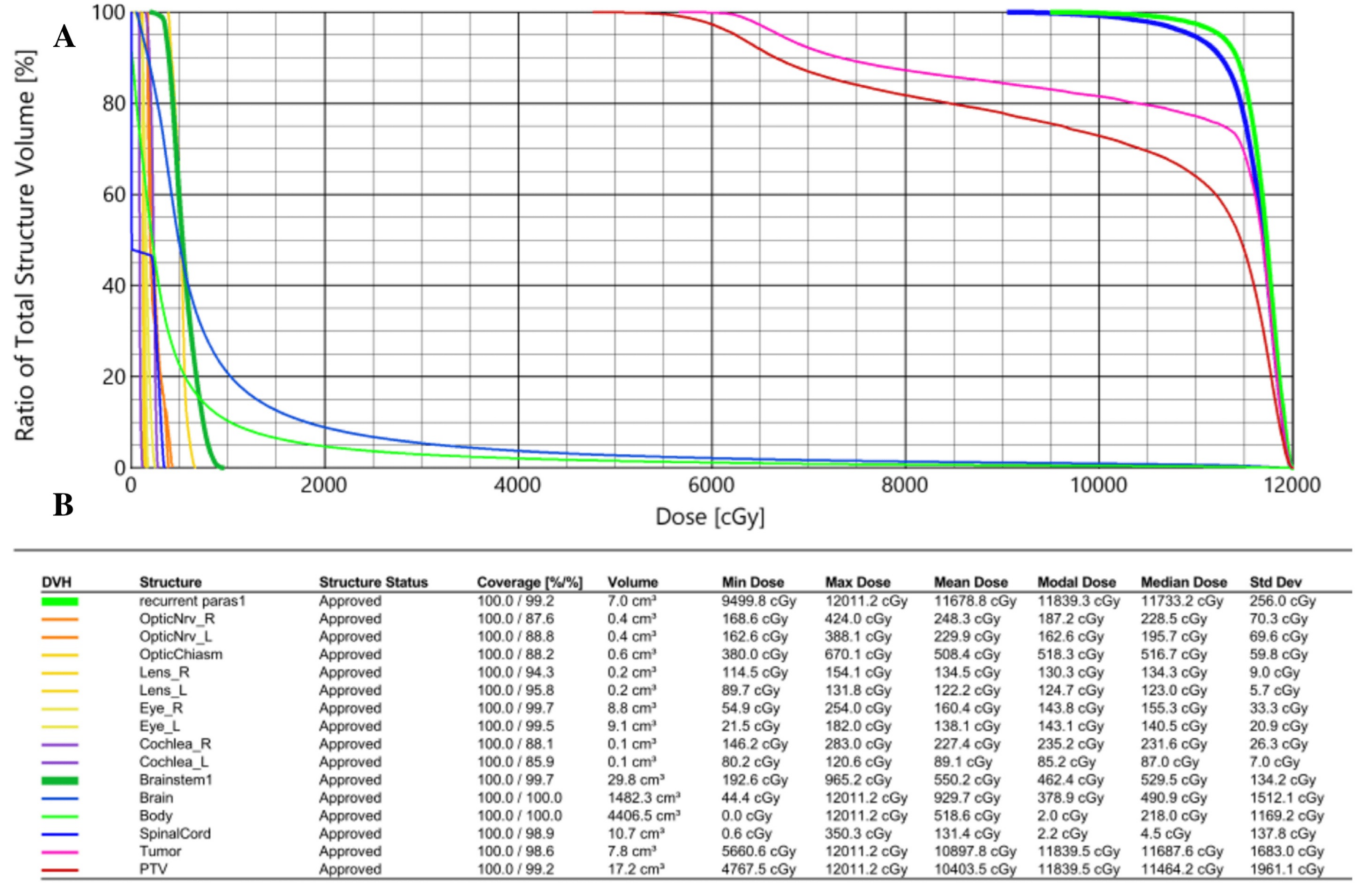
(A) Cumulative dose volume histogram (DVH) and (B) histogram legend.

Six years post intervention, serial MRI revealed progression of the left parietal convexity meningioma. The patient did not note any new neurological complaints associated with the growth of this tumor. A third left parietal craniotomy was performed with intraoperative monitoring. Her postoperative exam revealed no new neurological deficits. Postoperative pathology again confirmed grade I status, and genetic analysis revealed a new somatic NF2 alteration and an increase of the Ki-67 proliferative index to 12.7%. NF2 mutation detection was done via next-generation sequencing. Seven months postoperatively, while no recurrence was evident at the parietal site, the residual parasagittal tumor exhibited growth, with invasion into the superior sagittal sinus. The patient endorsed subjective word-finding difficulties and felt her memory was declining. Given the large size of the lesion, with a maximum dimension of 5.5 cm, in conjunction with her history of previous radiation to the anatomical region, fractionated SRT was used to decrease the risk of radionecrosis. The patient then received 55 Gy delivered in 22 fractions to achieve optimal control with minimized toxicity. The only acute radiation toxicity noted was grade 2 headaches and alopecia in the radiation field and grade 1 fatigue. Figures 1, 2 represent the composite radiation dose with isodose lines and a cumulative dose volume histogram, respectively.

The patient is currently doing well with no residual radiation toxicities, besides grade 2 alopecia in the radiation field. She denies any neurological symptoms, such as weakness, headaches, numbness, or changes in vision. According to multiple follow-up MRIs, the meningioma is currently stable, with the most recent MRI being eight months after salvage SRT. Table 1 summarizes the key clinical events.

**Table 1:** Key clinical events. SRT: stereotactic radiation therapy.

Date/timepoint	Event	Treatment	Outcome
Initial diagnosis (~12 years ago)	Initial diagnosis of grade I parafalcine vertex meningioma	Craniotomy	Initial recovery
10 years post initial surgery	Recurrence of left frontal meningioma, anterior to the previous craniotomy site	Craniotomy; pathology: Grade I, Ki-67: 1.3%	No evidence of recurrence
2 years post second craniotomy	Progression of left parasagittal meningioma	Fractionated linear accelerator-based stereotactic radiotherapy (SRT) (5400 cGy, 30 fractions)	Stable disease with mild symptoms (headaches, scalp discomfort)
2 years post SRT	New 7 mm recurrent left parietal meningioma found adjacent to the primary tumor	Single-fraction linear accelerator SRT (1500 cGy)	Lesion reduced in size; no toxicity
6 years post first SRT	Progression of left parietal convexity meningioma	Craniotomy; pathology: Grade I with NF2 mutation, Ki-67: 12.7%	No deficits postoperatively
7 months post third craniotomy	Growth of a parasagittal tumor with sagittal sinus invasion	Salvage SRT (55 Gy in 22 fractions)	Stable disease with grade 2 headaches and alopecia; grade 1 fatigue
8 months post salvage SRT	Latest follow-up MRI	Monitoring with MRI	Stable disease with no recurrence; no neurological symptoms

## Discussion

Here, we report on a case of what was initially diagnosed as a benign meningioma, which ultimately recurred with an aggressive clinical course. Even when these lesions follow their clinically stereotyped slow growth and infrequent recurrence, numerous longitudinal studies conducted over several decades have indicated that it is reasonable to expect a high recurrence rate with surgical GTR alone [9]. Notably, Pettersson-Segerlind et al. conducted a longitudinal study following grade I meningioma patients after surgical GTR. Over 25 years, the total recurrence rate was 47%, with 10 and 25-year mortality rates being 33% and 63%, respectively [9]. However, the consensus within the expert scientific community is that these lesions must be further characterized to adequately predict their clinical behavior and guide treatment intensity [9]. Consequently, some measure of controversy remains regarding adjuvant treatment even after GTR in these lesions.

There are numerous clinical parameters that have been found to correlate with more aggressive behavior amongst newly diagnosed grade I meningiomas. For instance, one retrospective study identified age >65 years, non-skull base location, and increased Ki-67 indices as predictors for atypical and malignant transformation in recurring meningiomas [10]. Another retrospective review further found that patients with non-skull base tumors who experience a recurrence were more likely than patients with skull base tumors to have higher-grade tumors at recurrence [11]. Interestingly, they found a cutoff of 4.5% for MIB-1, a separate marker for cell proliferation, to be a risk factor for recurrence [11,12]. Still, the utility of using MIB-1 as a predictor of recurrence is debated, as different cutoffs are used.

Another prognosticator of poor clinical outcomes in terms of local recurrence is the somatic loss of NF2, as seen in our patient. NF2 is a tumor suppressor gene on chromosome 22 that encodes the Merlin protein. Its loss activates oncogenic pathways leading to cell proliferation [1]. Deletion of this gene is found in 50-60% of meningiomas and 75% of atypical meningiomas [1]. An additional predictor of meningioma aggressiveness and recurrence is the proliferative index, defined by the immunohistochemical detection of Ki-67 antigen [13]. This supports findings in our patient, as she had an increase in Ki-67 index from 1.3% to 12.7% in the episode of recurrence, even though her tumor remained grade I throughout recurrence. Nowak et al. similarly found that Ki-67 indices >5% were associated with a two-fold increased risk of recurrence compared to Ki-67 indices <5% (27% vs. 13.1%) [13]. Interestingly, that study analyzed Ki-67 using a unique automated image analysis software that allows for reliable determination of Ki-67 values, minimizing inter-observer variability [13]. This opens a body of literature for using Ki-67 as a predictive factor for recurrence, regardless of tumor grade [1].

Regardless of the tumor’s anatomical location and ability to be surgically resected, grade I meningiomas have still led to recurrence in more aggressive forms. This may mean that surgery alone is not sufficient for disease control, especially for tumors located in anatomically challenging locations that make GTR difficult to achieve. For instance, the NRG/RTOG 0539 clinical trial highlighted that subtotally resected grade I meningiomas may benefit from adjuvant therapy, as these meningiomas had poor progression-free survival (PFS) with STR alone (three-year and five-year PFS rates were 83.1% and 72.7%, respectively) [14]. A promising study conducted by Soyuer et al. concluded that adjuvant RT after STR led to significantly reduced tumor progression, with a PFS rate of 91%, compared to a 38% PFS rate in those who received surgery alone [15].

Although optimal adjuvant RT techniques for recurrent meningiomas remain unclear, the 2021 European Association of Neuro-Oncology guidelines state that tumor recurrence should be treated with radiosurgery or FRT [8]. Our patient received SRT, which was associated with a significantly better PFS and overall survival (OS), as she is still experiencing disease stability. Overall, adjuvant RT can significantly reduce the progression of meningiomas, especially for those that are subtotally resected [14]. This further emphasizes that RT for meningioma recurrence can be a feasible option with good clinical outcomes.

Despite the benefits of reirradiation for recurrent meningiomas, it is often underutilized due to the risk of radiation toxicities and the concerns about the amount of radiation that surrounding tissue can tolerate [16]. However, a large multi-institutional retrospective study of 181 recurrent meningiomas was performed and found reirradiation to be a feasible treatment option with an acceptable toxicity profile. Specifically, after a median follow-up of 4.6 years, three-year PFS and OS rates were 51.6% and 72.5%, respectively, and no grade 5 toxicities were found [16]. The prevalence of radiation necrosis and peripheral edema was 9.9%, in line with previously published data [16]. Further, acute and late adverse events were reported in 21% and 16%, respectively, but these were correlated with a gross tumor volume (GTV) of ≥10cc at reirradiation. Another factor to consider is the time to reirradiation, as normal brain tissue dose tolerance is a limiting factor. After the first course of RT, recovery of occult injury is 50% at one year, 60% at two years, and 70% at three or more years [16]. This is important to understand, as details such as the timing of radiation can greatly affect the side effect profile. Aside from the neurological symptoms caused by our patient’s tumor, her radiation plan did not result in significant radiation toxicity, with only minimal headaches and expected scalp discomfort within the radiation field. A limitation of the study includes its retrospective nature.

## Conclusions

Although classified as benign, grade I meningiomas may exhibit recurrence and progression, particularly in anatomically complex regions that can only achieve subtotal resection, posing significant challenges in treatment planning. NF-2 alterations, alongside high Ki-67 proliferative indices and supratentorial location, have been associated with poorer prognosis in terms of PFS for patients presenting with grade I meningiomas. This case exemplifies the intricacies of managing recurrent grade I meningiomas, emphasizing the importance of multidisciplinary evaluation and tailored treatment approaches. Further, given the potential for late recurrences, surveillance imaging and long-term follow-up are necessary. Continued research in genomics and radiomics is critical to advance the classification and therapeutic strategies for meningiomas and to ultimately enhance patient outcomes.
